# YoLeTooth: A Unified Framework for Joint Tooth Segmentation and Periapical Lesion Detection in Panoramic Radiographs

**DOI:** 10.3390/jimaging12060272

**Published:** 2026-06-20

**Authors:** Gianmarco Scarano, Simone Agostinelli, Irene Amerini, Piero Papi

**Affiliations:** 1 ALCOR Lab, Department of Computer, Control and Management Engineering, Faculty of Information Engineering, Informatics and Statistics, Sapienza University of Rome, 00185 Rome, Italy; amerini@diag.uniroma1.it; 2 Department of Engineering and Science, Mercatorum University of Rome, Piazza Mattei 10, 00186 Rome, Italy; simone.agostinelli@unimercatorum.it; 3 Department of Oral and Maxillo-Facial Sciences, Sapienza University of Rome, 00161 Rome, Italy; piero.papi@uniroma1.it; 4 Clinic of General, Special Care, and Geriatric Dentistry, Center for Dental Medicine, University of Zürich, 8032 Zurich, Switzerland

**Keywords:** deep learning, computer vision, medical image segmentation, periapical lesion detection, teeth segmentation

## Abstract

Chronic periapical periodontitis is a persistent inflammatory disease characterized by progressive bone destruction around the tooth apex. Manual radiographic detection of these lesions is subjective and time-consuming, highlighting the need for automated diagnostic tools. This paper presents a unified deep learning framework for joint tooth segmentation and periapical lesion detection in panoramic radiographs. Our approach employs a joint process: first, a deep learning model identifies and segments individual teeth according to standard dental numbering systems, while a second one detects periapical lesions within the tooth regions obtained from the segmentation outputs in the first stage. The framework incorporates an advanced loss function (Powerful IoU v2) to improve bounding-box regression accuracy and a spatial association mechanism to map detected lesions to specific teeth based on geometric overlap analysis. Our proposed tooth segmentation model achieves an mAP@50 of 97.7% and a mean Dice coefficient of 93.5%, while the periapical lesion detector reaches an mAP@50 of 91.9%. Furthermore, our region-of-interest approach yields a 3.49× computational speedup, averaging 0.1589 s per radiograph when compared to full-image processing. Trained exclusively on open-source datasets, this reproducible framework achieves explicit tooth-to-lesion mapping, providing an efficient and practical tool for periapical lesion screening.

## 1. Introduction

Periapical periodontitis is a chronic inflammatory condition characterized by slow, progressive, and osteoclast-dependent bone resorption around the apex of the tooth. The primary agent involved in the development of this condition is the microbial infection of the root canal system [1]. Periapical periodontitis is one of the most commonly occurring diseases in the global community, affecting a significant proportion of adults, being also one of the leading reasons for tooth extraction if left untreated, as a result of the continuous irritations [2]. Aside from its localized destructive effect in the oral cavity, chronic periapical periodontitis has gained more recognition for its strong impact in contributing to this systemic disease. The local inflammatory burdens have the potential to become a circulating factor, facilitating the exacerbation of systemic disorders. In particular, there is a relationship with Diabetes Mellitus, meaning that diabetic patients have a higher risk of suffering from periapical lesions during the course of the disease and experience delayed and poorer healing outcome following endodontic therapies [3,4,5].

**Figure 1:**
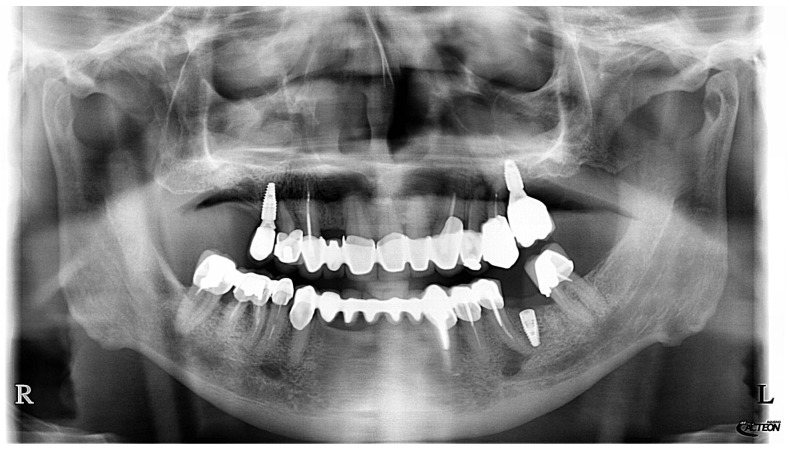
A visualization of a periapical lesion in a panoramic radiograph. The lesion appears as a darker radiolucent area (indicating decreased bone density) located at the apex of the tooth root. This darker appearance is due to the bone resorption and inflammatory process which is characteristic of periapical periodontitis.

Radiographic assessment via 2D panoramic radiographs, denoted as orthopantomograms (OPTs), is a key diagnostic tool in the clinical evaluation of periapical periodontitis, since the typical periapical radiolucencies, which surround the root apex, are easily identifiable in these images. The presence of periapical radiolucencies is an indication that the disease comprises approximately 75% of radiolucent jaw lesions [5]. Even if 2D imaging is the main diagnostic tool, largely due to their easy availability and low dose of radiation to the patient, it is fundamentally limited by 2D projection of 3D anatomical structures [6]. The Periapical Index (PAI) was developed in 1986 to provide clinicians with a method of severity standardization during radiographic interpretation of these lesions, as well as indicating the appropriate endodontic treatment plan, such as root canal therapy [7]. The PAI ranges from one to five, where a PAI score of one indicates healthy apical tissues and a PAI score of five indicates severe periapical periodontitis with acute exacerbation. An example of a periapical lesion in a panoramic radiograph is shown in Figure 1.

Although OPTs are widely available and the PAI scoring system is well established, manual clinical examination and manual interpretation of these scans can be a challenging and time-consuming task for dental experts. The visual detection of periapical lesions is subjective and dependent on the clinician’s experience, and is often complicated by low image contrast, anatomical overlap of adjacent dental structures, and imaging artifacts [6,8]. The limitations of early-stage apical periodontitis being clinically asymptomatic and radiographically undetectable until a considerable cortical bone mineral loss is present [9] leads to an increased risk of delayed diagnosis, high inter-examiner variability and inconsistent treatment choices, especially in large-scale screening settings.

To fill this research gap, we propose YoLeTooth, a unified DL framework that follows a novel two-stage pipeline design to jointly perform tooth segmentation and periapical lesion detection. Trained on open-source datasets to facilitate reproducibility, our approach first localizes individual dental structures to define an anatomically relevant region of interest and then conducts targeted, region-aware lesion detection within these boundaries. Unlike generic detectors, YoLeTooth provides a clear tooth-to-lesion mapping using the FDI notation system, marking a shift from simple bounding-box pathology detection to a localized, tooth-level diagnosis. By automatically associating each detected lesion with a specific tooth via geometric overlap analysis, our unified approach improves the speed, reliability, and clinical interpretability of the final diagnosis compared to methods that treat these tasks independently. With this architecture, we demonstrate that reliable and efficient diagnostic systems can be developed using a limited amount of publicly available data, offering a practical tool for automated endodontic screening. Our hypothesis is that by first establishing anatomically precise tooth boundaries and then constraining lesion detection to those regions, a unified framework can reliably associate each detected pathology with its corresponding tooth, while reducing the computational weight of full-image processing.

## 2. Related Works

Researchers have conducted several studies in the area of Medical Imaging with Machine Learning (ML) and deep learning (DL) techniques for detection, classification and segmentation of diseases through images [10,11]. Specifically, researchers have applied those techniques to segment dental structures (to isolate individual teeth or regions of interest) and classify pathologies (such as caries, bone loss, or periodontitis). These two tasks are often treated independently, but in practice, they are tightly coupled, as accurate classification of dental diseases often requires precise tooth localization.

In particular, in the dentistry domain, numerous studies have classified and detected dental diseases using different imaging modalities (2D and 3D) including periapical radiographs, cone beam computed tomography (CBCT), and panoramic radiographs [12,13,14]. For example, Lee et al. [15] proposed a DL framework using a pre-trained GoogLeNet Inception v3 [16] network for the automatic detection of dental caries from periapical radiographs, with 89.0% (95% CI: 80.4–93.3%), 88.0% (95% CI: 79.2–93.1%) and 82.0% (95% CI: 75.5–87.1%) accuracy in detecting premolar, molar and combined premolar and molar caries respectively. In [17], the authors exploited a seven-layer feed-forward CNN to predict the periodontal bone loss (PBL) from panoramic dental radiographs, with a performance score of 0.81 as mean accuracy.

Eftimie et al. [18] tried to map dental pathologies to individual teeth with object detection models like YOLOv11, achieving a segmentation accuracy of 0.645 (mAP50-95), which could cause misalignment in complex scenarios, as the accurate mapping depends heavily on the accurate segmentation of tooth boundaries. In order to address this, the work presented here employs an optimized segmentation backbone that improves such precision as described in Section 4.1.

Other works, such as [19], focused on segmenting and extracting wisdom teeth starting from radiographs via the software MATLAB, while Koch et al. [20] exploited an ensemble of U-Net networks through test-time augmentation (TTA) for segmenting teeth from pantomograms. More recent works focused on the use of U-Net for teeth mask segmentation via panoramic radiographs, such as [21], which achieved an accuracy score of 0.98, but without providing any information about the teeth numbers. Finally, due to the introduction and the innovation of the Transformer architecture [22], Kanwal et al. [23] proposed an attention-based model for segmenting teeth from panoramic images, achieving an accuracy of 0.97 points (with Average Precision sitting at 0.98 ± 0.4), at the expense of high computational complexity.

For periodontitis prediction, DeNTNet [24] employs a transfer learning approach using four Neural Networks to predict periodontal bone loss (PBL) in panoramic radiographs. However, PBL refers to marginal bone loss (also known as MBL), a clinical feature of periodontitis, while our work focuses on periapical lesions, which are evaluated through the Periapical Index (PAI) and indicate apical periodontitis. For this reason, the task addressed by DeNTNet is not directly comparable to ours. Moreover, the study does not provide any details regarding inference times, the most critical factor for real-world applications and, furthermore, there are very limited methods that combine tooth segmentation with lesion detection within a common framework that is required to map pathological findings to individual teeth. An exception can be found in [25], which presents a DL framework for stage classification of periodontitis through the application of two segmentation masks: PBL (periodontal bone loss) and CEJ (Cemento-Enamel Junction), which are utilized to compute Radiographic Bone Loss (RBL). However, this approach differs significantly from ours in several key aspects: it focuses on marginal bone loss assessment rather than periapical lesion detection, which relies on manually annotated expert data that are not publicly accessible, further restricting its reproducibility, and does not provide explicit lesion-to-tooth mapping through spatial association methods. Furthermore, it lacks computational efficiency considerations and inference time analysis.

Several studies have been conducted for automating the diagnosis of periapical lesions using different types of radiographic modalities. In particular, Ba-Hattab et al. [26], Celik et al. [27] and Song et al. [28] successfully employed Convolutional Neural Networks (CNNs) to detect and classify periapical radiolucencies on 2D panoramic radiographs. Issa et al. [29] evaluated intraoral periapical X-rays and assessed a commercial U-Net-based AI tool, demonstrating high diagnostic accuracy. However, they stated that detection models alone may misclassify some lesions due to anatomical overlaps and due to not being trained on different imaging modalities. Recently, Chau et al. [30] moved towards 3D-based imaging and segmentation models by presenting CBCT-SAM, an AI method based on the Segment Anything architecture for periapical lesion detection, while Fu et al. [31] and Hadzic et al. [32] applied dedicated 3D CNNs to assess these lesions on cone-beam computed tomography (CBCT) scans. While these architectures demonstrate strong diagnostic metrics for isolated lesion identification, they share limitations that can hinder their clinical deployment.

First, the vast majority of these models are trained and evaluated on private, proprietary, or highly limited datasets. For example, studies by Ekert et al. [33] and Endres et al. [34] relied on relatively small and inaccessible data samples, a constraint that precludes the verification of results, restricts reproducibility, and prevents fair comparisons across different architectures. Furthermore, existing approaches focus almost exclusively on single-task, isolated pathology detection, meaning simply drawing a bounding box or mask around a lesion. Studies by Altukroni et al. [35] and Bayrakdar et al. [36] successfully detected and segmented periapical lesions on panoramic radiographs, respectively, but they lacked the structural integration required to simultaneously segment the surrounding dental anatomy and explicitly map the detected lesion to a specific tooth using standard clinical numbering (e.g., the Fédération Dentaire Internationale (FDI) notation system). Finally, many of these studies lack computational efficiency considerations and inference time analysis, which are strict prerequisites for real-world clinical applications. Consequently, despite the growing interest in dental AI, no previous work has combined tooth segmentation and periapical lesion detection into a unified framework that geometrically associates lesions with specific teeth.

Beyond CNN-based approaches, recent architectural paradigms have explored alternatives to overcome the limitations of both convolutions and self-attention mechanisms. The emergence of State-Space Models (SSMs), particularly Mamba [37], offers linear-complexity global context modeling (O(N)) as an alternative to the quadratic complexity ($O(N^{2})$) of Transformers. Hybrid CNN–Mamba architectures, such as HCMNet [38], combine CNN encoders for local feature extraction with Mamba-based decoders for long-range dependencies, demonstrating improved performance in medical image segmentation tasks. Foundation models such as the Segment Anything Model (SAM) have also been adapted for medical imaging through parameter-efficient fine-tuning and knowledge distillation [30,37]. While these paradigms show promise for dense-prediction tasks, our framework follows a different design structure: rather than a single model for pixel-level segmentation, we decompose the diagnostic process into two specialized stages (tooth segmentation followed by region-aware lesion detection) that operate independently. This task-specific, ROI-driven approach achieves competitive accuracy with lower computational overhead with just 0.16 s of inference time per radiograph, while providing structured clinical output (FDI tooth-to-lesion mapping) that is not inherently available from general-purpose segmentation models.

## 3. Materials and Methods

### 3.1. Framework Overview

**Figure 2:**
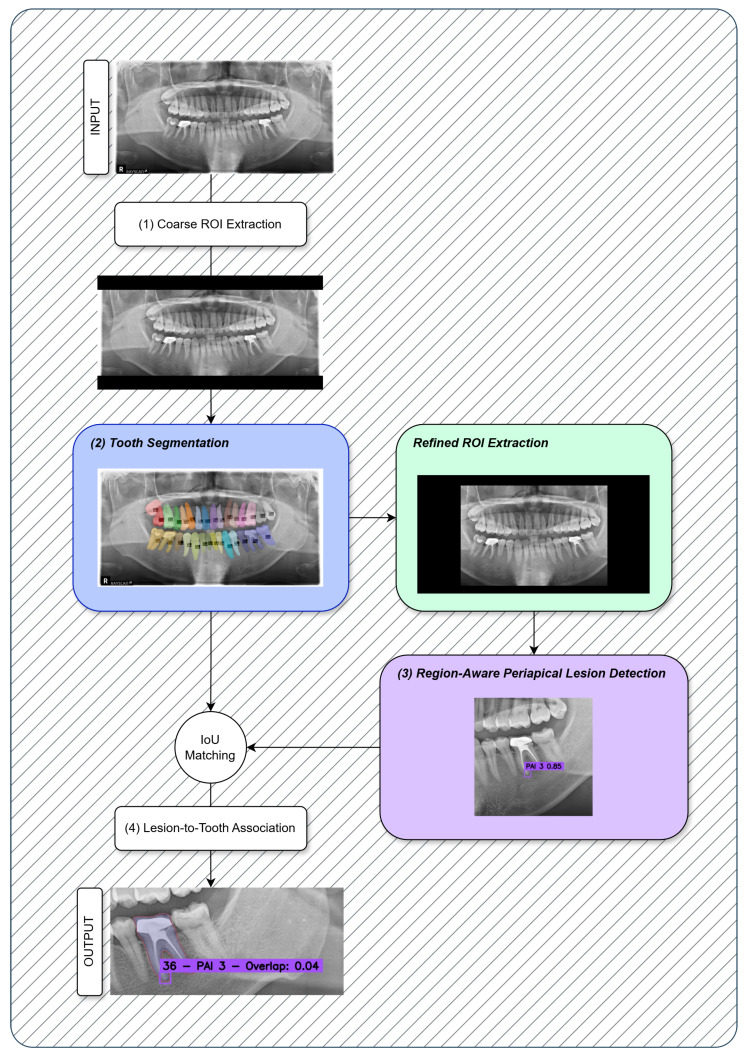
Overview of the proposed YoLeTooth framework. The pipeline consists of four main stages: (1) coarse ROI extraction from the OPT, (2) tooth segmentation, (3) region-aware periapical lesion detection based on previous segmentation masks, and (4) lesion-to-tooth association within the refined region. The final output includes IoU spatial association between detected lesions (3) and corresponding teeth (2) with overlap measurements.

The proposed YoLeTooth framework has four main steps: (1) coarse region of interest (ROI) extraction, (2) tooth segmentation, (3) region-aware periapical lesion detection, and (4) lesion-to-tooth association, where we employ an Intersection-over-Union (IoU) spatial association mechanism for tooth-to-lesion mapping. Figure 2 shows the framework uses a cascaded methodology where each step takes advantage of the output of the previous step, with the final output being a joint diagnosis that links detected lesions to specific teeth.

The first stage consists of a coarse ROI extraction in order to shift the focus on the dental region of the OPT. The second stage makes use of a CNN-based segmentation model to identify and segment individual teeth within that region, generating up to 32 various segmentation masks portraying the entirety of the adult dentition. Specifically, they range from 0 to 31 for normal teeth, along with a separate class 32 specific for the supernumerary teeth. The third stage uses these ROI-based segmentation masks to create an optimized ROI for the next lesion detection phase. The fourth stage employs a CNN-based detection model to identify periapical lesions within this refined ROI. Those lesions are classified into three classes (0, 1, and 2) corresponding to PAI level 3, 4, and 5, respectively. Finally, the framework is able to provide and link any associated lesion to a specific tooth by analyzing the spatial overlap between the detected lesions and the single teeth.

In detail, we propose a unified architecture that introduces several improvements for research studies. Firstly, the modular design of the segmentation and detection steps enables their replacement and optimization independently with more advanced or customized models, allowing for further and future enhancements without re-engineering the overall system from scratch. While our current methodology utilizes modified YOLO-based [39] models for both segmentation and detection, this generalizable framework is able to accommodate other architectures like U-Net [40] for segmentation or RF-DETR [41] for detection. In fact, the overall design is made flexible and expandable such that the inclusion of more diagnostic modules or the extension to other imaging modalities is possible with a straightforward integration. Secondly, ROI-based detection reduces computational overhead while maintaining detection accuracy by limiting the operation to anatomically significant regions, reducing the input area by an average of 25% compared to a full-image processing, resulting in an overall speedup, which will be further investigated in Section 4.2. For both detection and segmentation tasks, an improved version of the bounding-box loss has been implemented, named Powerful IoU v2 (PIoUv2) loss [42], which enhances the accuracy of bounding-box predictions by incorporating positional information, further discussed in Section 3.3. Finally, the implicit tooth-to-lesion mapping provides meaningful information that surpasses generic lesion detection approaches.

### 3.2. Stage 1: Coarse ROI Extraction

The first stage of the proposed framework is the extraction of regions of interest (ROIs) from the panoramic radiograph, which is used as input. We crop the OPT to a rectangular bounding box to capture the whole dentition, which is typically found in the middle of the image within a 50–70% margin on the vertical axis, since there can be cases of bad or misaligned image acquisition, where the teeth are not precisely centered in the image. The final output is a cropped rectangular region of interest ($B_{\mathrm{ROI}}$) containing the general dental area.

Let σ be the ROI factor; then, the ROI is defined as:

(1) $$B_{\mathrm{ROI}}=\left(x,y,w,h\right)=\left(0,\frac{H\cdot (1-\sigma )}{2},W,H\cdot \sigma \right)$$

where

(x,y) is the top-left corner of the bounding box;
*w* is the width of the bounding box;
*h* is the height of the bounding box;
*W* is the width of the original OPT image;
*H* is the height of the original OPT image;

This coarse, preliminary ROI extraction is a simple yet effective method to reduce the computational overhead in the subsequent stages by focusing on the relevant anatomical structures, ignoring unnecessary background and irrelevant regions of the image. This is particularly useful in dental radiographs, where the teeth are typically located in the central part of the image [43].

### 3.3. Stage 2: Tooth Segmentation

In this stage, we refined a YOLO-Seg [44] model, training it on a specific tooth segmentation dataset (cf. Section 3.6.1) to adapt pre-trained features and weights for dental structure recognition. We trained the model to highlight each dental structure and produce segmentation masks for every tooth identified in the input cropped ROI ($B_{\mathrm{ROI}}$) from Stage 1.

The quantity of masks produced by the model differs, as it is based on the dental structure and anatomy of the patient, which involves taking into account missing teeth, impacted teeth, or supernumerary teeth above the usual number. This phase outputs different segmentation masks, each corresponding to an individual tooth number, following the FDI convention [45], thus, facilitating their identification.

To further improve the accuracy of the final output masks, we integrated the Powerful IoU v2 (PIoUv2) loss function into the model, replacing the conventional Intersection-over-Union (IoU) loss function. The PIoUv2 loss overcomes drawbacks of traditional IoU measures by adding a penalty term taking into account the positional relation between predicted and ground-truth bounding boxes. When compared to the normal IoU loss, which merely takes the overlapping area into consideration, PIoUv2 involves the spatial position information; thus, it is especially useful for small object detection and localization refinement in crowded scenes where occluded objects might be present (e.g., overlapping or dental crowding). For this reason, this kind of loss is valuable for periapical lesion detection and tooth segmentation, as accurate lesion localization and proper tooth structure boundaries are necessary for a correct model evaluation.

The PIoUv2 loss is defined as:

(2) $$L_{PIoUv2}=1-\left(IoU-L_{v2}\right)$$

where IoU represents the standard Intersection-Over-Union metric, and $L_{v2}$ is the PIoUv2 penalty term defined as:

(3) $$L_{v2}=3\cdot x\cdot e^{-x^{2}}\cdot L_{v1}$$

with x=q·Λ, where $q=e^{-P}$, and Λ=1.3 is an empirically determined scaling factor found through multiple experiments. The positional penalty *P* encodes the relative displacement between bounding boxes:

(4) $$P=\frac{1}{4}\left(\frac{dw_{1}+dw_{2}}{|w_{2}|}+\frac{dh_{1}+dh_{2}}{|h_{2}|}\right)$$

where $dw_{1}$, $dw_{2}$, $dh_{1}$, and $dh_{2}$ are the absolute differences between the minimum and maximum coordinates of the ground-truth and predicted boxes in the horizontal and vertical directions, respectively. Finally, the base penalty term $L_{v1}$ is calculated as:

(5) $$L_{v1}=1-e^{-P^{2}}$$

The segmentation masks produced in this stage have two primary functions: they outline each tooth’s contour and provide a spatial reference, in terms of refined ROI, for the subsequent region-aware periapical lesion detection stage.

### 3.4. Stage 3: Region-Aware Periapical Lesion Detection

The *region-aware lesion detection* stage is divided into two sub-stages. The first sub-stage involves the computation of a refined region of interest (ROI) from the tooth segmentation masks generated during Stage 2. The second sub-stage consists in passing this ROI to the detection model for the final periapical lesion identification.

The generation of the ROI starts by calculating the bounding box for all the teeth from their segmentation masks. The overall ROI is then obtained by considering the minimum and maximum coordinates across all individual bounding boxes, which then define the smallest rectangle that contains all segmented teeth. This approach, instead of processing the entire OPT, which might contain irrelevant anatomical structures and thereby reducing computational overhead, allows the detection model to focus on areas which are more likely to contain lesions, which are the ones surrounding the segmented teeth.

For this matter, let the bounding box of the *i*th tooth be:

(6) $$B_{i}=\left(x_{i},y_{i},w_{i},h_{i}\right)$$

where $\left(x_{i},y_{i}\right)$ denotes the top-left corner of the bounding box, and $w_{i}$, $h_{i}$ its width and height, respectively.

The global ROI bounding box $B_{\mathrm{global}}$ is computed as:

(7) $$\begin{array}{cccc} x_{\min} & =\min_{i=1}^{n}x_{i} & y_{\min} & =\min_{i=1}^{n}y_{i} \end{array}$$

(8) $$\begin{array}{cccc} x_{\max} & =\max_{i=1}^{n}\left(x_{i}+w_{i}\right) & y_{\max} & =\max_{i=1}^{n}\left(y_{i}+h_{i}\right) \end{array}$$

(9) $$\begin{array}{cccc} w_{\mathrm{global}} & =x_{\max}-x_{\min} & h_{\mathrm{global}} & =y_{\max}-y_{\min} \end{array}$$

where *n* represents the total number of segmented teeth available in the OPT.

To identify periapical lesions that spread outside the tooth into adjacent alveolar and periodontal tissue, we further expand the global ROI to capture clinically relevant pathological areas without superfluous computation by removing irrelevant regions.

The expansion is controlled by horizontal and vertical scale factors α and β:

(10) $$\Delta_{v}=\left\lfloor h_{\mathrm{global}}\cdot \alpha \right\rfloor ,\;\Delta_{h}=\left\lfloor w_{\mathrm{global}}\cdot \beta \right\rfloor$$

where α,β∈[0,1] are coefficients determined empirically.

The final dilation ROI $B_{\mathrm{expanded}}$ is bounded by the image size (W,H):

(11) $$\begin{array}{c} x_{\exp}=\max\left(0,x_{\min}-\Delta_{h}\right), \end{array}$$

(12) $$\begin{array}{c} y_{\exp}=\max\left(0,y_{\min}-\Delta_{v}\right), \end{array}$$

(13) $$\begin{array}{c} w_{\exp}=\min\left(W-x_{\exp},w_{\mathrm{global}}+2\Delta_{h}\right), \end{array}$$

(14) $$\begin{array}{c} h_{\exp}=\min\left(H-y_{\exp},h_{\mathrm{global}}+2\Delta_{v}\right) \end{array}$$

We then pass the expanded bounding box as input to the periapical lesion detection model (YOLO-Det). This model is a refined YOLO-Det [46], trained on a dataset containing periapical lesion annotations with bounding-box labels (cf. Section 3.6.1). This solid foundation enables the model to recognize the characteristic radiographic patterns of periapical pathology. To further improve bounding-box localization, we incorporated the Powerful IoU v2 (PIoUv2) loss [42], as previously explained in Section 3.3. This loss enhances localization accuracy by penalizing discrepancies in the position, shape, and scale of bounding boxes, which is particularly beneficial for detecting small and irregularly shaped periapical lesions. The model processes this refined ROI to identify and classify periapical lesions, producing bounding boxes with associated PAI levels for each detected lesion.

### 3.5. Stage 4: Lesion-to-Tooth Association

The integration of segmentation and detection results occurs through a spatial association process. As input, this stage takes the tooth segmentation masks (from Stage 2) and the detected lesion bounding boxes (from Stage 3). The process then matches each lesion to its specific tooth by calculating the Intersection over Union (IoU). This association ensures each detected lesion is matched with the anatomy of the most pertinent tooth, thus providing meaningful diagnostic outcomes. The final output includes the lesion’s PAI class, the tooth’s FDI number, and the overlap measurement.

#### 3.5.1. Tooth and Lesion Representation

We model each segmented tooth as a polygon $T_{i}=\left\{\left(x_{1},y_{1}\right),\ldots ,\left(x_{n_{i}},y_{n_{i}}\right)\right\}$, and we calculate its area through the use of the Shoelace formula [47], as follows:

(15) $$A\left(T_{i}\right)=\frac{1}{2}\left|\sum_{j=1}^{n_{i}}\left(x_{j}y_{j+1}-x_{j+1}y_{j}\right)\right|$$

where $x_{j}$ and $y_{j}$ are the coordinates of the $T_{i}$’s vertices, and $n_{i}$ is the number of vertices.

We represent every periapical lesion $L_{j}$ as a bounding box specified by its corner coordinates $\left(x_{\min},y_{\min}\right)$ and $\left(x_{\max},y_{\max}\right)$.

#### 3.5.2. Overlap and Association

For tooth polygon $T_{i}$ and lesion polygon $L_{j}$, we compute the intersection area $I_{i,j}=T_{i}\cap L_{j}$ using geometric overlay operations. The area of this region, $A(I_{i,j})$, is used to compute the overlap fraction:

(16) $$Coverage_{i,j}=\frac{A(I_{i,j})}{A(T_{i})}$$

We associate the lesion with tooth $T_{i}$ if ${\mathrm{Coverage}}_{i,j}\geq \tau$, where τ∈[0,1] is a predefined threshold. The obtained association set is:

(17) $$A=\left\{\left(i,j,c_{i,j}\right)|{\mathrm{Coverage}}_{i,j}\geq \tau \right\}$$

where $c_{i,j}$ represents the confidence score of the detected lesion $L_{j}$. For clarity, the complete lesion-to-tooth association procedure is summarized in Algorithm 1.
**Algorithm 1:** Lesion-to-tooth spatial association.
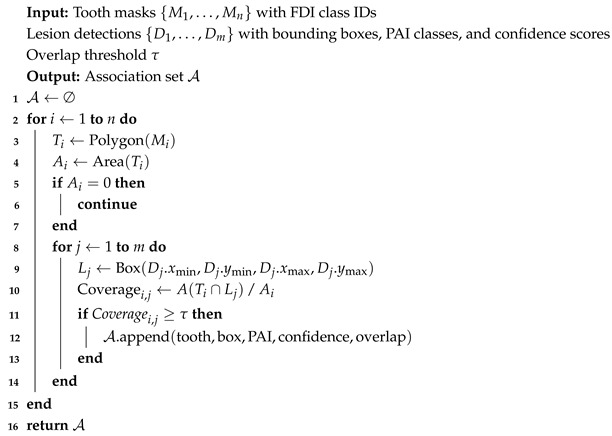


The proposed framework generates outputs that include both the identified lesions (classified into 3 classes: 0, 1, and 2, corresponding to PAI-levels 3, 4, and 5, respectively), the respective tooth numbers (ranging from 0 to 31 for normal teeth, with class 32 specific for supernumerary teeth), and the corresponding overlap value between any detected lesion and its associated tooth. This spatial correlation enables automatic lesion matching to the most probable involved teeth according to the geometric overlap.

### 3.6. Implementation Details

#### 3.6.1. Datasets and Training Setup

We trained and validated the proposed framework exclusively on two open-source datasets specially designed for tooth segmentation and periapical lesion detection in panoramic X-ray images, supporting reproducibility. Code implementation can be made available on request.

The tooth segmentation dataset was derived from the publicly accessible DualLabel Dataset [48], consisting of a total of 2066 annotated panoramic radiographs with pixel-level segmentation masks for every tooth. The annotations were performed by a team of five medical professionals: two postgraduate students and two dentists with over five years of experience conducted the primary labeling, while a senior dentist with over 15 years of experience reviewed the annotations. The dataset allows for up to 32 various tooth classes (including supernumerary teeth class 91) and adopts the FDI notation system, which gives each tooth a unique number so that the lesions can be easily mapped and identified to individual teeth. The authors of the dataset state that patient data privacy and confidentiality are kept anonymous by clearing the images of various personally identifiable information.

The publicly released periapical lesion detection dataset [49] consists of 3926 images with bounding-box annotations established by three experienced dentists (each with more than 5 years of clinical practice), classifying periapical lesions based on the Periapical Index (PAI) scoring system. The augmented version of the dataset, which was used for this work, makes use of random scaling (0.8–1.2×), random rotation (−90° to 180°), mirroring (horizontal, vertical, and combined), and noise injection, for a total of 17,004 images collected between January 2016 and March 2021, with a resolution of 2444×1292 pixels and 2316×1292 pixels. We trained the model on a workstation with an RTX 5000 Ada Generation GPU (32 GB) and Intel Xeon Gold 6338 CPU, with all input images normalized to 1280×1280 pixels. During inference, we first localized and cropped a dental region of interest (ROI) to restrict analysis to anatomically relevant structures for tooth segmentation and periapical lesion detection; this allowed us to reduce the input tensor to 640×640 pixels, decreasing inference time. The distribution of periapical lesions across PAI severity levels is shown in Table 1. The dataset exhibits a natural class imbalance, with PAI 3 lesions representing the majority (61.2%), followed by PAI 4 (30.1%) and PAI 5 (8.6%). We did not apply explicit class balancing (e.g., oversampling or class weighting) as a preprocessing step, as the natural distribution reflects real-world clinical prevalence where early-stage lesions are more common than advanced ones.

#### 3.6.2. Pipeline Configuration and Parameters

YoLeTooth employs several carefully selected parameters to balance computational efficiency and diagnosis accuracy. The inference pipeline operates with confidence thresholds $\theta_{seg}=0.50$ for tooth boundary detection and $\theta_{det}=0.25$ for periapical lesion detection. The initial ROI extraction is performed with a vertical factor σ=0.75, covering the middle 75% of the image’s height where teeth are typically present.

We calculated the segmentation-based ROI expansion by using (14), with a vertical expansion factor α=0.30 and a horizontal expansion factor β=0.05 to gain a more extensive coverage of periapical regions while maintaining computational efficiency. The spatial association step employs a coverage threshold of τ=0.04 as shown in (17), meaning that detected periapical lesions are associated with a particular tooth when the intersection area covers at least 4% of the tooth’s segmentation mask area. We validated these parameters (σ, α, β, τ) through extensive ablation studies on a held-out validation set, optimizing the trade-off between computational efficiency and diagnostic accuracy; in fact we correctly associated 90.32% of the lesions with their respective teeth using these settings. In total, out of 3399 images containing 5857 lesions, 5288 fell inside the ROI expansion and 146 were partially cut due to the intersection of the detected lesion with the image border. We also carried out an iterative grid-search parameter test, which showed that the system maintained more than 90% of inclusion rate until α dropped below a value of 0.15.

#### 3.6.3. Evaluation Metrics

For the segmentation task, the following metrics were considered: bounding-box metrics (tooth localization) as well as mask-based (accurate tooth boundary definition) metrics such as precision, recall, Mean Average Precision at IoU threshold 0.5 (mAP50), and Mean Average Precision averaged over IoU thresholds 0.5 to 0.95 (mAP50-95). For U-Net, the Mean Dice Coefficient (mDice) and Mean Intersection-over-Union (mIoU) are reported as the main segmentation metrics, since mAP is not applicable for pixel-level segmentation tasks, hence we adapted the YOLO final predictions to align with those metrics. For detection evaluation metrics, precision, recall, mAP50, and mAP50-95 were taken into account for the three PAI severity classes to determine the performance of the model to localize and classify periapical lesions, whereas for pipeline performance, inference time was the key metric of interest.

## 4. Results

We designed various experiments to assess the performance of our proposed system, YoLeTooth, for individual teeth segmentation as well as for the detection of periapical lesions. In our segmentation experiment, we report bounding-box metrics (mAP50, mAP50-95) and mask-based metrics (mDice, mIoU), while in our detection experiment, we provide mAP50 and mAP50-95 for three severity levels of PAI.

### 4.1. Teeth Segmentation Results

We conducted the segmentation experiments using our proposed model, YOLO-Seg. The specific choice of this model and the detailed rationale for selecting it as the final proposed model is thoroughly explained in Section 4.3, where we present comprehensive hyperparameter optimization for loss functions, model architectures and learning rates. Overall, when selecting the best overall configuration for the tooth segmentation task, we gave priority to the computational efficiency and training stability of the model, as well as driving specific attention to the mAP@50-95 metric, as it provides a more cohesive evaluation of model performance and generalization across varying IoU thresholds.

#### Comparison with State-of-the-Art Methods

Since most of the available dental segmentation datasets are exclusive and confidential, a direct comparison of our results to published methods on their identical test sets is difficult. To overcome this limitation, we rebuilt some of the state-of-the-art approaches and trained them on our dataset under similar experimental settings, ensuring a fair comparison.

Table 2 shows a performance comparison between our method and state-of-the-art methods, highlighting that our proposed model YOLO-Seg (YOLOv12-turbo-m-seg [50]) achieved competitive performance in terms of mAP@50-95, as well as in mAP@50. It achieved an mAP@50 of 0.9773 and an mAP@50-95 of 0.7903, trained for 300 epochs with the PIoUv2 loss, as explained in Section 3.3, AdamW optimizer, a momentum of 0.9, weight decay of 0.0001, lr of 0.00027, a batch size of eight, and an image size of 1280 × 1280 pixels.

Furthermore, YOLOv9e [48] achieved a marginally better performance of the mAP@50 metric, with 0.9801 points, but an mAP@50-95 of 0.7853, at the expense of requiring 60.4 M parameters, compared to our proposed model, which achieved almost the same precision but with only 22.3 M parameters, a reduction of 2.7× in model size. Our proposed method also achieved the highest mAP@50-95 of 0.7903, outperforming YOLOv9e by 0.0050 points, demonstrating that the efficiency gains did not come at the expense of overall segmentation quality.

YOLOv8m [51] achieved an mAP@50 of 0.9736 and an mAP@50-95 of 0.7835, while the DentSeg [52] (YOLO-based) framework reached an mAP@50 of 0.9733 and an mAP@50-95 of 0.7785.

Other traditional CNN-based architectures showed much lower performance. Mask R-CNN [53], commonly used in medical imaging, managed only an mAP@50 of 0.7620 and mAP@50-95 of 0.5990, highlighting the difficulty of using two-stage detectors in this field. ResNeSt-50 [53], another well-known architecture, reached an mAP@50 of 0.9590 and mAP@50-95 of 0.7590, while the baseline YOLOv5m [54] model achieved an mAP@50 of 0.9591 and mAP@50-95 of 0.7193.

**Figure 3:**
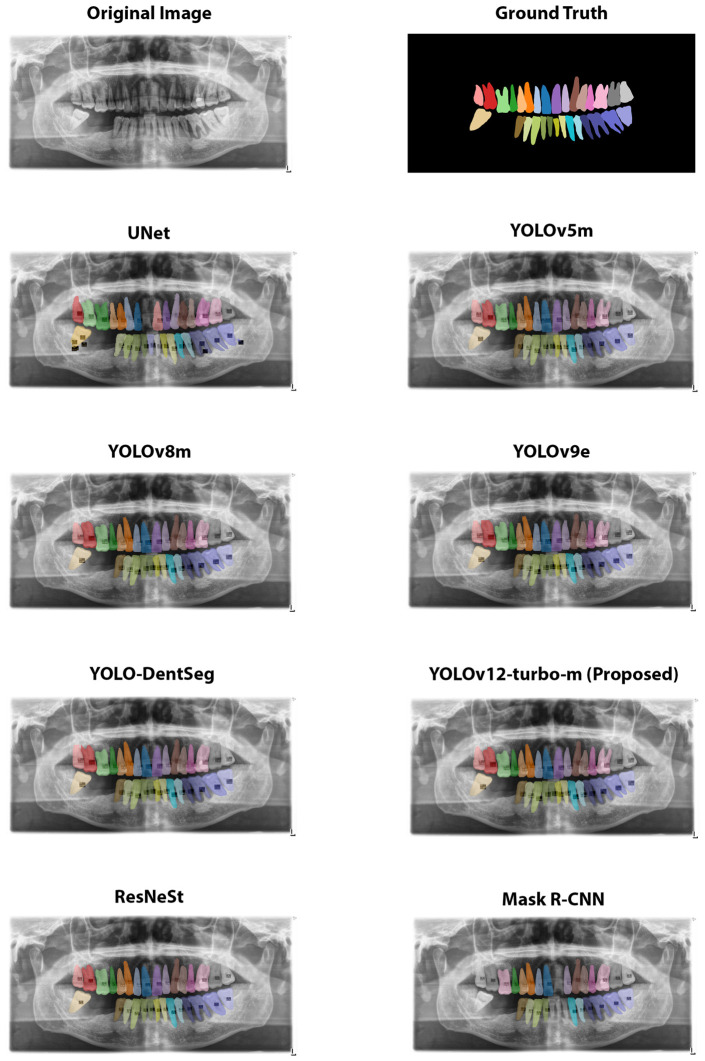
Segmentation results comparison between a test image, our proposed model, and different SOTA methods.

**Figure 4:**
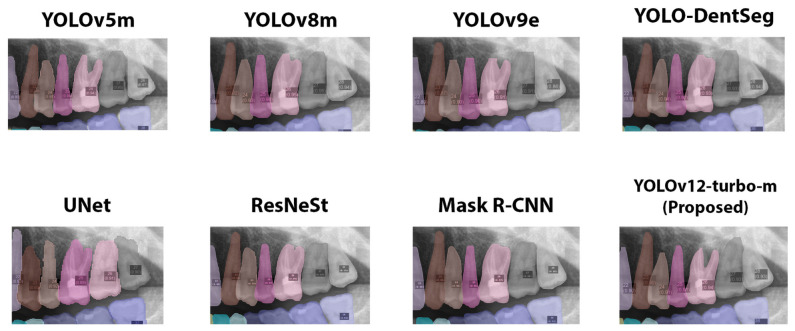
Detailed segmentation results comparison focusing on tooth number 26. Our proposed model produces cleaner masks than other evaluated SOTA methods.

Finally, we performed experiments using the U-Net architecture, a well-documented and widely used model for the task of image segmentation, and the results are shown in Table 3. Among the different variants of the U-Net architecture used for the experiments, the best configuration used the following hyperparameters: AdamW optimizer, *batch size* of 12, image size of 512×512 pixels, 200 training epochs, $lr=1\times 10^{-3}$, and weight decay = $5\times 10^{-4}$. This particular variant of the U-Net architecture achieved a validation mDice of 0.8803 and mIoU of 0.8063. The proposed model performed much better than the U-Net model in terms of segmentation masks, achieving a validation mDice score of 0.9354 and *mIoU* score of 0.8784, a difference of 5.5 and 7.2 percentages points, respectively. Figure 3 shows the various segmentation masks produced by the different SOTA models and the proposed YOLOv12-turbo-m-seg model, which produces much more accurate and cleaner results. This is further shown in Figure 4, which illustrates our model’s superior precision in capturing tooth boundaries for tooth number 26 when compared to the noisier and less precise masks from other SOTA methods.

### 4.2. Periapical Lesion Detection Results

For periapical lesion detection, we used our proposed model, YOLO-Det. We validated this choice by comparing it with other architectures like YOLO versions 8, 9, and 11, and transformer-based alternatives like RF-DETR.

The detection experiments used two different training settings to analyze different training aspects:

- **Standard**: The model was trained with default hyperparameters and YOLO’s native image augmentation for robustness.
- **Lightweight**: This configuration substituted the standard Convolutional Layer with the GhostConvolution Layer [55] to lower computational costs.

**Figure 5:**
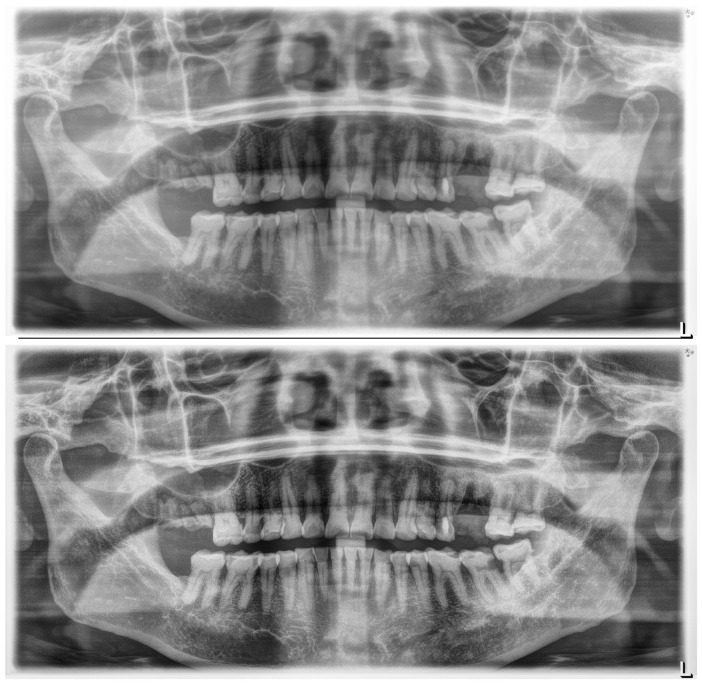
Examples of original (**top row**) and enhanced (**bottom row**) images from the tooth segmentation dataset after applying the proposed preprocessing pipeline. The enhancement techniques include sharpening, CLAHE, and Gaussian blur to improve image quality and contrast for better model training.

We also investigated an enhanced preprocessing pipeline, which included sharpening, Contrast-Limited Adaptive Histogram Equalization (CLAHE), and Gaussian blur to enhance image quality before training, as illustrated in Figure 5. Models were trained for 80 to 300 epochs, monitoring model accuracy.

Table 4 summarizes the periapical lesion detection analysis. The proposed YOLO-Det (YOLOv12-m [50]) outperformed all other methods with 0.9194 mAP@50 and 0.7794 mAP@50-95 on only 20.1 M parameters, using the *Standard* configuration. The proposed PIoUv2 loss proved effective, raising mAP@50 by 0.6 percentage points (from 0.913 to 0.9194) and mAP@50-95 by 1.3 points (from 0.766 to 0.7794) compared to the standard IoU loss. This confirms PIoUv2 is beneficial for both segmentation (as shown in Section 4.1) and detection, particularly for accurate localization of small, spatially precise lesions. Our proposed model used SGD optimization with *lr* = 1 × 10^−2^, batch = 6, epochs = 300, warmup epochs = 5, image size = 1280×1280 and an early stopping with a value of patience = 30.

Although other state-of-the-art models, such as Faster R-CNN [56], could have been employed, YOLO’s single-stage architecture provides faster inference speed without compromising on quality [57]. As depicted in Table 4, our validation of the RF-DETR (Detection Transformer) model indicated relatively competitive results (0.9123 mAP@50, 0.6563 mAP@50-95) but required longer training times. This justifies selecting the latest YOLO model, which provides a strong balance between accuracy and computational efficiency.

The *enhanced* preprocessing configuration yielded mixed results: YOLOv12-m achieved 0.9023 mAP@50 and 0.7403 mAP@50-95, demonstrating that additional preprocessing did not consistently improve detection performance. YOLOv11-m showed 0.8553 for mAP@50 and 0.6161 for mAP@50-95, while the large model, YOLOv11-x, showed 0.8522 mAP@50 and 0.6222 mAP@50-95. Note that the large model was not remarkably better than its medium-sized counterpart. This indicates that a larger model capacity is not always beneficial for this task.

YOLOv9-c model performed consistently well, achieving 0.8863 mAP@50 and 0.6603 mAP@50-95 under *standard* training, demonstrating its architectural robustness. On the other hand, our attempts to make architectures more lightweight revealed performance degradation. For example, the *lightweight* configuration of YOLOv12-m with GhostConvolution achieved lower scores (0.7260 mAP@50, 0.4800 mAP@50-95). Similarly, the YOLOv12-turbo-m with these layers showed lower accuracy (0.7861 mAP@50, 0.5131 mAP@50-95). This suggests general lightweight techniques may work poorly in medical imaging, which requires precise detection of subtle, small lesion features.

**Figure 6:**
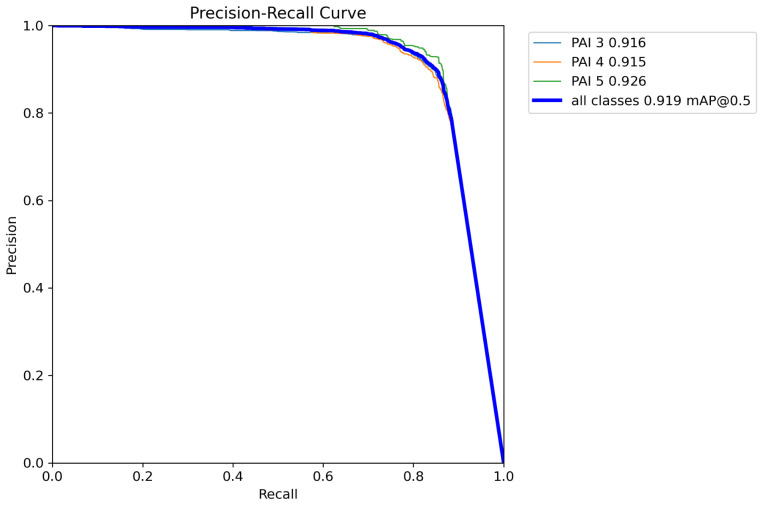
Precision–recall curves for periapical lesion detection across all three PAI severity classes. Each curve shows the trade-off between precision and recall as the confidence threshold varies. The macro-averaged curve (bold blue) demonstrates consistent performance across classes with an mAP@50 of 0.919.

To further characterize the detection performance across confidence thresholds, we present the precision–recall curves for all three PAI severity classes in Figure 6. The curves demonstrate that the model maintains high precision (above 0.90) across a wide range of recall values for all classes, with a macro-averaged mAP@50 of 0.919. This indicates consistent detection performance regardless of lesion severity.

Finally, Table 5 reports the computational performance of our ROI-based method, outlined in Section 3.4, comparing full-image and ROI-based inference times on a single OPT image. In the ROI-based method, we first cropped an anatomically relevant dental ROI (reducing the processed field of view by about 25% on average) and then fed the network with a smaller input tensor of 640×640 pixels (while the full-image baseline used 1280×1280). For CNN-based models, the forward-pass cost scales approximately with the number of input pixels (H×W) [58,59], so halving the side length (1280→640) reduces the spatial compute by ∼4×. We benchmarked the pipeline on both GPU (RTX 5000 Ada) and CPU (Intel Xeon Gold 6338) to provide a comprehensive view of computational requirements.

The ROI-based speedup is substantially larger on GPU (up to 4.16×) than on CPU (up to 1.33×), as GPU inference benefits more from reduced memory bandwidth and higher parallelism with smaller input tensors. On CPU, the ROI extraction overhead and sequential memory access patterns attenuate the speedup, though the pipeline still processes each radiograph in approximately 0.55 s.

### 4.3. Hyperparameter Optimization for the Segmentation Phase

This section presents our model selection and hyperparameter tuning procedure, rather than a methodological ablation study. In order to optimize our model of tooth segmentation, we conducted an in-depth search over four phases, considering (1) loss functions, (2) model sizes, (3) learning rates, as well as (4) warm-up and scheduling optimizations. Finally, we conducted additional experiments for hyperparameter optimization. In all those experiments, we applied the *enhanced* data augmentation pipeline, as previously explained in Section 4.2. Several other segmentation architectures are included as baselines for context.

#### 4.3.1. Loss Functions and Parameter Efficiency

The first phase of our optimization study evaluated the impact of different loss functions on model performance. To enhance the accuracy of bounding-box regression for the segmentation task, we incorporated the Powerful IoU v2 (PIoUv2) loss function, which is particularly suitable for precise tooth structure boundary outlining, as described in Section 3.3, and compared it against the conventional Intersection-over-Union (IoU) loss, specifically CIoU.

Table 6 presents the Phase 1 results, comparing PIoUv2 loss with CIoU loss in order to confirm its effectiveness. Results demonstrate performance improvements achieved by integrating this loss in the segmentation models, where the addition of the PIoUv2 loss in the YOLOv12-turbo-x-seg variant resulted in an mAP@50 of 0.9785 points and an mAP@50-95 of 0.7899 points, outperforming the CIoU variant (mAP@50 of 0.9771 and mAP@50-95 of 0.7895). Significantly, this loss integration also obtained promising results for medium-sized models, as observed in the YOLOv12-turbo-m-seg (22.3 M parameters) model, with an mAP@50 of 0.9800 and an mAP@50-95 of 0.7859, compared to CIoU’s mAP@50 of 0.9749 and mAP@50-95 of 0.7850, reaching the best score for mAP@50 for this phase. For YOLOv8m-seg, the PIoUv2 loss led to an mAP@50 of 0.9758 and an mAP@50-95 of 0.7871, improving over CIoU’s mAP@50 of 0.9736 and mAP@50-95 of 0.7835. Finally, the same behavior is visible in the case of the YOLOv11-l model [60], with an mAP@50 of 0.9788 and an mAP@50-95 of 0.7926, showing improvements over standard CIoU (mAP@50 of 0.9783 and mAP@50-95 of 0.7886). With these experiments, we point out that these consistent improvements across different model sizes validate PIoUv2 as a general key architectural enhancement for tooth segmentation tasks in dental radiographs, rather than being specific to a particular model.

Phase 2 tried diverse model sizes to get the best trade-off between accuracy and computational cost. From Table 7, YOLOv12-turbo-m-seg achieved the best performance for mAP@50 with a score of 0.9800, 2.8× smaller than its YOLOv12-turbo-x-seg counterpart, while the YOLOv11-m version variant was also competitive, showing a strong result regarding mAP@50-95 with a score of 0.7902. Small models such as YOLOv12-turbo-n-seg achieved reasonable performance (mAP@50 of 0.9716) with the smallest number of parameters (2.7 M), making it an efficient option. We included the nano-sized models in this phase specifically to evaluate how models with a very low parameter count performed in terms of accuracy compared to their larger counterparts, while other sizes such as small, medium, and large were employed to analyze how accuracy was affected by increasing the number of parameters, providing a clear view of the trade-off between model complexity and performance.

Phases 1 and 2 provided some useful insights for the final model selection. Although the YOLOv11-l architecture achieved the highest mAP@50-95 during Phase 1 (0.7926), the latest version of the YOLO architecture, 12-turbo, demonstrated competitive performance with the ‘x’ variant reaching a close second (0.7899), only 0.0027 points lower. In addition, it is evident that there is a key trade-off here for model size in Phase 2. Our proposed model, YOLOv12-turbo-m-seg, shows us that it has the highest mAP@50 (0.9800) with a notable difference in model sizes, 2.8× smaller than its ‘x’ variant and other competing models, including YOLOv11-l, by having just 22.3 M parameters.

#### 4.3.2. Learning Rate and Scheduler Optimization

Based on findings in Phases 1 and 2, we analyzed different learning rate settings in Phase 3 (Table 8). In that phase, we varied the learning rate value from 0.0005 to 0.01. The proposed model, YOLOv12-turbo-m-seg, with $lr_{0}=0.001$, performed best, with an mAP@50 of 0.9800 and mAP@50-95 of 0.7859. This suggests that a moderate learning rate value has a beneficial effect in learning or in converging for this particular task. In the fourth phase of the optimization studies, Warmup and Scheduler, respectively, had values ranging from one to five, the cosine learning rate ($cos_{lr}$) was considered as a Boolean value, and the final learning rate was fixed to 0.01. Table 9 summarizes these results. The results show that our proposed model recorded the best score for mAP@50 (warmup=3, $cos_{lr}=true$) with 0.9800. Furthermore, the same model but with cosine learning rate deactivated reached an mAP@50 of 0.9728, with a difference of 0.0072 points. YOLOv8-m-seg (warmup=3, $cos_{lr}=true$) showcased the best performance for mAP@50-95 with 0.7871 points.

Based on the results from the various optimization phases, the choice of the YOLOv12-turbo-m-seg model with *PIoUv2* loss, $lr_{0}=0.001$, warmup=3, and $cos_{lr}=true$ was validated as the proposed model, achieving an mAP@50 of 0.9800 and mAP@50-95 of 0.7859. This configuration served as the baseline for the subsequent fine-tuning analysis.

With the previous phases completed, we proceeded to test the previously selected model by investigating hyperparameter optimizations such as optimizer selection, image resolution, batch size, weight decay, and dropout to check whether these modifications had a positive benefit in the entire learning process. In all the following experiments, we kept all the remaining parameters constant based upon previous phases of optimization, varying the specific hyperparameter under examination to check its contribution to model performance.

#### 4.3.3. Optimizer Comparison

The first fine-tuning analysis examined the choice of optimizer and its associated hyperparameters. We compared four different optimizers, SGD with varying momentum values, Adam, NAdam, and RMSProp, to determine which one provided the best optimization setup for this task. We tested momentum values between 0.9 and 0.95, and batch sizes ranging from two to four, as these parameters significantly influence the convergence trajectory and final model accuracy. We fixed the input image resolution at 1280×1280 pixels. Table 10 presents these results, demonstrating that SGD with a batch size of two had the highest performance (mAP@50 of 0.9800), but a slightly higher momentum initialization benefited the mAP@50-95 score, reaching 0.7877 points. Adam and NAdam had weaker performance, while RMSProp performed worse, indicating that those optimizers may be less suitable for this task.

#### 4.3.4. Image Resolution Analysis

Following the optimizer selection, we proceeded to examine the impact of input image resolution on model performance. Modern DL models often benefit from higher-resolution inputs, especially for tasks requiring fine-grained feature detection, such as tooth structure boundary delineation. We evaluated image resolutions of 640×640, 1024×1024, 1280×1280, and 1600×1600 pixels, slightly varying the batch size and the optimizer selection to gain insights into their interactions. The rationale behind those ranges was to assess the trade-off between resolution-driven accuracy improvements and computational resource requirements. When auto was chosen as the optimizer, the framework automatically selected the best optimizer based on the dataset characteristics. From Table 11, it is clear that higher resolutions, such as 1600×1600, led to the highest performance in terms of mAP@50-95 at 0.7978, highlighting that a higher image resolution is beneficial in detailed structure segmentation of dental radiographic images, although it presents a limitation in terms of increased required processing power. Image sizes such as 640×640 led to lower accuracies of around 0.7094 and 0.7151 in mAP@50-95. Despite this, the model’s performance when using an input size resolution of 1280×1280 was still competitive and achieved the highest mAP@50 of 0.9800, inducing a better speed–accuracy trade-off.

#### 4.3.5. Batch Size Optimization

The batch size is a critical hyperparameter that affects both training dynamics and computational memory requirements. We examined batch sizes of two, four, eight, and 10 using different optimizer hyperparameters to understand how batch size interacted with optimizer choice. In general, smaller batch sizes (such as two) are often considered to make gradients noisier yet can be helpful in getting out of local minima, while larger batch sizes (such as eight or 10) make it possible to get more precise estimates of the gradients. From Table 12, batch-size hyperparameter tuning provided minor boosts in performance at smaller batch sizes, where the SGD optimizer at a batch size of two provided the best mAP@50 value of 0.9800, while auto optimizer at a batch size of four provided the best value of 0.7896 in terms of mAP@50-95. This experiment indicates that while smaller batch sizes enhance peak accuracy (mAP@50), moderate-sized batches may actually yield better overall performance (mAP@50-95) estimations. An increase in batch sizes above eight was not beneficial and did not bring further improvements.

#### 4.3.6. Weight Decay and Regularization Strategy

Weight decay is a regularization method that prevents large weight values in order to train simpler models that generalize well. We tested weight decay regularizers of 0.0001, 0.0005, and 0.001 with early stopping patience of 30 and 50, respectively, while fixing the SGD optimizer at $lr_{0}=0.001$. We selected these ranges based on common practices in DL: typical weight decay values fall between $10^{-4}$ and $10^{-3}$, while early-stopping patience values typically range from 20 to 50 epochs. Moreover, while performing most of our experiments, we used a value of 10 for the close mosaic augmentation parameter (Close Mosaic), but we also tested a value of five to analyze its effect as a regularization factor. This close mosaic augmentation is a regularization technique that enables mosaic augmentation in a region around the object of interest, thereby enabling the network to pay attention to all features. We can observe from Table 13 that a lower weight decay of 0.0005 led to better IoU generalization with an mAP@50 of 0.9800, indicating that higher regularization techniques worked better in this particular task, but the experiment with a weight decay of 0.0001 better balanced generalization across IoU thresholds, resulting in more stable and reliable performance in practice. Moreover, an increased value of 0.001 in weight decay led to a slight improvement in mAP@50-95 of 0.7889, thereby indicating a trade-off between peak accuracy and overall performance across IoU thresholds. Finally, the close mosaic augmentation parameter, when set to 10, achieved an mAP@50 of 0.9800, outperforming the same model with a value of five (mAP@50 of 0.9728), indicating that stronger augmentation helped the model to generalize better.

#### 4.3.7. Dropout Regularization

Finally, we investigated the effects of dropout regularization, which randomly deactivates neurons during training to prevent co-adaptation and improve generalization. We applied a dropout rate of 0.0 (no dropout), 0.1 (light regularization), and 0.2 (medium regularization) to our proposed YOLOv12-turbo-m-seg model. We present our findings in Table 14, illustrating that a dropout rate of 0.0 or zero regularization produced the best possible balance with an mAP@50 of 0.9800. In addition, more heavily regularized networks with a dropout rate of 0.2 recorded a decrement in performance with an mAP@50 of 0.9723, inferring that overly regularized networks might inhibit the model’s ability to learn intricate features. An appropriately balanced regularization factor of 0.1 recorded an mAP@50-95 of 0.7896, which was the highest in this analysis.

This detailed analysis validates our model selection, confirming that the YOLOv12-turbo-m-seg configuration with a weight decay value of 0.0001, as detailed in Table 13, is the best-suited model for this task, thereby justifying the choice introduced in Section 4.1. Summarizing, the optimization studies demonstrated that: (1) PIoUv2 loss is effective in dental segmentation in radiographs, performing better than CIoU; (2) medium-sized models have the optimal efficiency–accuracy trade-off; (3) careful learning rate tuning is very important for stability and convergence; (4) SGD with appropriate momentum outperforms other optimizers in this task; and (5) higher image resolution improves performance at the cost of increased computational complexity.

#### 4.3.8. Reproducibility and Robustness Analysis

To assess the stability and reproducibility of our reported results, we conducted two complementary analyses which consisted in performing five independent training runs of the proposed segmentation model (YOLOv12-turbo-m-seg with PIoUv2 loss) using different random seeds (123, 456, 789, 1024, 426), while keeping all hyperparameters, data augmentation settings, and the training/test split fixed. The results are summarized in Table 15.

All metrics exhibited a coefficient of variation below 0.3%, confirming that the reported performance is not dependent on a specific random weight initialization.

Furthermore, as part of the model selection procedure described in Section 4.3, we systematically evaluated 37 segmentation configurations across varying learning rates, batch sizes, image resolutions, optimizers, and regularization strategies. Of these, 89% (33/37) achieved mAP@50-95 ≥ 0.77, and 97% (36/37) exceeded 0.71 on the same metric. This broad coverage of the hyperparameter space demonstrates that the model consistently achieves high performance across a wide range of settings, rather than relying on a narrowly tuned configuration.

## 5. Discussion

This study presented YoLeTooth, a novel unified DL framework that jointly performs tooth segmentation and periapical lesion detection in panoramic radiographs for automatic periapical lesion screening. Our two-stage pipeline fuses YOLO-based models with the Powerful IoU v2 loss function, which combines strong performance with computationally efficient ROI-based preprocessing.

Through a set of multiple experiments organized across multiple phases, we investigated loss function validation, model size exploration, learning rate optimization, optimizer selection, image size effects, batch size tuning, weight decay configurations, and dropout strategies. The proposed method achieved mAP@50 of 0.9800 and mAP@50-95 of 0.7859 for tooth segmentation, surpassing state-of-the-art methods when trained on the same dataset. For detection, the proposed approach scored 0.9194 mAP@50, surpassing RF-DETR and other variants. The ROI-based approach gave a 3.49x total speedup over the full-image processing, achieving a high-speed inference time with an average of only 0.1589 s per radiograph.

**Figure 7:**
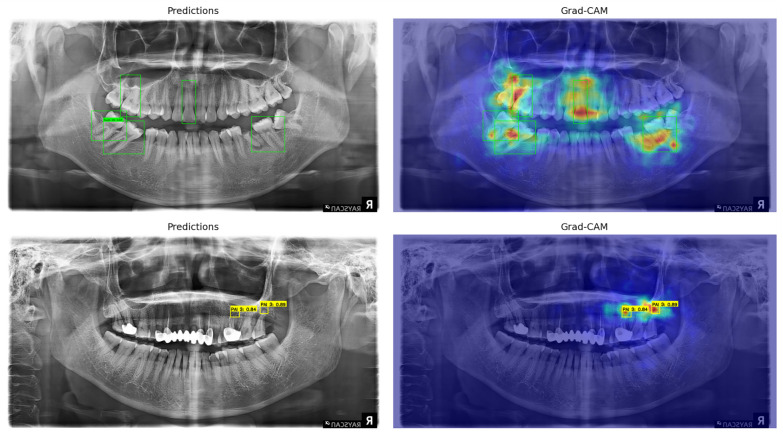
A visualization of Grad-CAM activation maps for both the segmentation (**top**) and detection (**bottom**) stages of the proposed YoLeTooth framework. The (**left**) column shows the original panoramic radiograph with overlaid bounding boxes for detected lesions, while the (**right**) column presents the corresponding Grad-CAM heatmaps, highlighting the regions that contributed most to the model’s predictions. The detection stage heatmaps focus on periapical areas, while the segmentation stage heatmaps emphasize tooth boundaries and anatomical features. For visual clarity, only a subset of predicted bounding boxes is displayed in the segmentation stage.

To provide insight into the decision-making process of our models and increase trustworthiness, we conducted a Grad-CAM (Gradient-weighted Class Activation Mapping) analysis [61] for both the segmentation and detection stages. Figure 7 illustrates the ROI-aware Grad-CAM visualizations for a representative panoramic radiograph. For the detection stage, the activation maps demonstrate that the model predominantly focuses on the periapical regions surrounding tooth apexes, correctly highlighting the radiolucent areas corresponding to periapical lesions across different PAI severity levels. The segmentation stage visualizations confirm that the model attends to tooth boundaries and anatomical structures when producing segmentation masks, which inherently start from bounding-box localization.

One of the most important contributions of this work is the tooth-lesion mapping, which offers insightful diagnosis information by directly linking identified periapical lesions to actual tooth numbers, numbered according to the FDI notation system, ensuring that the correct tooth undergoes appropriate treatment. In a real clinical scenario, a proper list regarding affected teeth and their corresponding lesions would be an addition for dentists, facilitating any possible medical documentation and diagnostic process, as well as reducing possible mistakes. Furthermore, we envision this tool as an assistant tool, which is able to generate a summary table with tooth-lesion mapping, allowing for a faster revision by experts. Our research integrates openly available datasets to promote reproducibility and ease of access for the research community, an important feature not always available in other research. The state-of-the-art comparisons demonstrate that our optimized configuration with PIoUv2 loss achieves robust performance for dental segmentation from panoramic radiographs.

## 6. Conclusions

In this work, we presented YoLeTooth, a unified deep learning framework for joint tooth segmentation and periapical lesion detection in panoramic radiographs, trained exclusively on open-source datasets. The framework represents a reproducible and clinically oriented screening tool that shifts the paradigm from isolated pathology detection to structured, tooth-level diagnosis via the FDI notation system. The framework’s modular design allows for straightforward integration of advanced architectures and extension to additional diagnostic modules.

Since the current model is based exclusively on a categorized PAI dataset, its use in a real-world clinical setting needs to be carefully validated. Hence, we plan to address this as future work by conducting evaluations and validation from experts in the field such as radiologists and periodontists to confirm the generalizability and clinical applicability of the framework. It is worth noting that our two-stage pipeline introduces a potential source of cascading errors: if the segmentation stage misses a tooth, any periapical lesion on that tooth cannot be detected. Given the segmentation recall of 97.37% of our model, at most 2.63% of lesions (~154 out of 5857 from the dataset) are potentially missed due to segmentation failure. This upper bound is conservative, as the actual number is lower due to the fact that not all missed teeth contain lesions. Additionally, adding very early-stage lesions (PAI 1-2) as well as correctly segmenting any possible external prosthetics (bridges, crowns and braces) from the OPTs, would make the model more robust, as those cases are often found in real clinical scenarios and represent a current limitation of our framework. Finally, another interesting feature enhancement involves focusing on increasing robustness for mixed dentition cases. While the current ROI parameters work well for the adult dentition, the system’s architecture allows for flexibility through parameter re-tuning. By adjusting the vertical margins (α,β) and lowering the refinement threshold τ, the model could better accommodate mixed dentition, considering the non-standard vertical dentition positioning often found in pediatric radiographs.

## Figures and Tables

**Table 1:** Distribution of periapical lesions across PAI severity levels in the overall dataset.

PAI Level	Number of Lesions	Percentage (%)
3	3587	61.2%
4	1765	30.1%
5	505	8.6%
Total	5857	100%

**Table 2:** Comparison of teeth segmentation results with state-of-the-art methods (trained on the open-source DualLabel dataset).

Model	Parameters	mAP50	mAP50-95
YOLOv12-turbo-m-seg (Proposed)	22.3 M	0.9773	**0.7903**
YOLOv9e [48]	60.4 M	**0.9801**	0.7853
YOLOv8m [51]	27.2 M	0.9736	0.7835
YOLO-DentSeg [52]	25.2 M	0.9733	0.7785
ResNeSt-50 [53]	33.0 M	0.9590	0.7590
YOLOv5m (Baseline) [54]	**21.8 M**	0.9591	0.7193
Mask R-CNN [53]	29.0 M	0.7620	0.5990

Results in bold indicate the best performance, while underlined results indicate the second-best performance.

**Table 3:** U-Net experimental results on the teeth segmentation task. The proposed YOLOv12-turbo-m-seg model outperforms U-Net in both mDice and mIoU metrics.

Model	Opt.	Batch	Size	Epochs	LR	Mom.	Weight D.	mDice	mIoU
YOLOv12-turbo-m-seg (Proposed)	AdamW	8	1280	300	2.7 $\times \;10^{-4}$	0.9	1 $\times \;10^{-4}$	**0.9354**	**0.8784**
U-Net	AdamW	12	512	200	1 $\times \;10^{-3}$	—	5 $\times \;10^{-4}$	0.8803	0.8063
U-Net	AdamW	16	512	200	1 $\times \;10^{-3}$	—	1 $\times \;10^{-4}$	0.8572	0.7702
U-Net	SGD	2	1024	200	1 $\times \;10^{-2}$	0.99	1 $\times \;10^{-4}$	0.8392	0.7412
U-Net	SGD	2	512	200	1 $\times \;10^{-2}$	0.99	1 $\times \;10^{-4}$	0.7251	0.5820

Results in bold indicate the best performance.

**Table 4:** Comparison of detection models on the periapical lesion detection. The proposed YOLOv12-m model achieves the best performance in both mAP@50 and mAP@50-95 metrics.

Model	mAP@50	mAP@50-95	Params	Epochs	Enh. ^†^
YOLOv12-m (Proposed)	**0.9194**	**0.7794**	20.1 M	300	No
RF-DETR-Base	0.9123	0.6563	29.0 M	250	Yes
YOLOv12-m	0.9023	0.7403	20.1 M	275	Yes
YOLOv9-c	0.8863	0.6603	25.5 M	80	No
YOLOv11-m	0.8553	0.6161	20.1 M	80	No
YOLOv11-x	0.8522	0.6222	56.9 M	80	No
YOLOv8-m	0.8443	0.6343	25.9 M	80	No
YOLOv8-x	0.8342	0.6363	68.2 M	80	No
YOLOv12-turbo-m	0.7861	0.5131	19.6 M	300	No
YOLOv12-m + Ghost	0.7260	0.4800	**17.4 M**	300	Yes

^†^ Enh.: Whether the enhanced preprocessing pipeline (sharpening, CLAHE, Gaussian blur) was applied during training. Results in bold indicate the best performance.

**Table 5:** Computational performance of full-image and ROI-based approaches on GPU (RTX 5000 Ada) and CPU (Intel Xeon Gold 6338).

Task	GPU (RTX 5000 Ada)			CPU (Xeon Gold 6338)		
	Full (s)	ROI (s)	Speedup	Full (s)	ROI (s)	Speedup
Segmentation	0.4120	0.0991	4.16×	0.5234	0.3987	1.31×
Detection	0.1420	0.0597	2.38×	0.1511	0.1135	1.33×
Combined	0.5541	0.1589	3.49×	0.6370	0.5497	1.16×

**Table 6:** Phase 1: Validation of loss function. The proposed model achieves the best performance in mAP@50.

Model	Loss	mAP50	mAP50-95
YOLOv12-turbo-m-seg	CIoU	0.9749	0.7850
YOLOv12-turbo-m-seg (Proposed)	PIoUv2	**0.9800**	0.7859
YOLOv12-turbo-x-seg	CIoU	0.9771	0.7895
YOLOv12-turbo-x-seg	PIoUv2	0.9785	0.7899
YOLOv8m-seg	CIoU	0.9736	0.7835
YOLOv8m-seg	PIoUv2	0.9758	0.7871
YOLOv11l-seg	CIoU	0.9783	0.7886
YOLOv11l-seg	PIoUv2	0.9788	**0.7926**

Results in bold indicate the best performance.

**Table 7:** Phase 2: Exploration of different model sizes. The proposed model achieves the best performance in mAP@50.

Model	Parameters	mAP50	mAP50-95
YOLOv11n-seg	2.8 M	0.9707	0.7711
YOLOv11s-seg	10.0 M	0.9748	0.7843
YOLOv11m-seg	22.3 M	0.9758	**0.7902**
YOLOv11l-seg	27.6 M	0.9748	0.7896
YOLOv8n-seg	3.2 M	0.9744	0.7712
YOLOv8s-seg	11.8 M	0.9765	0.7828
YOLOv8m-seg	27.2 M	0.9758	0.7871
YOLOv8l-seg	45.9 M	0.9707	0.7883
YOLOv12-turbo-n-seg	**2.7 M**	0.9716	0.7659
YOLOv12-turbo-m-seg (Proposed)	22.3 M	**0.9800**	0.7859
YOLOv12-turbo-l-seg	27.6 M	0.9742	0.7880
YOLOv12-turbo-x-seg	62.0 M	0.9785	0.7899

Results in bold indicate the best performance.

**Table 8:** Phase 3: Optimization of the learning rate value. The proposed model is marked with ^†^.

Model	*lr*_0_	lrf	mAP50	mAP50-95
YOLOv12-turbo-m-seg ^†^	0.001	0.01	**0.9800**	0.7859
YOLOv12-turbo-m-seg	0.0005	0.01	0.9787	0.78617
YOLOv11-m-seg	0.0002	0.01	0.9763	0.78619
YOLOv12-turbo-m-seg	0.001	0.1	0.9758	0.7811
YOLOv8-m-seg	0.0002	0.01	0.9758	**0.7871**
YOLOv12-turbo-m-seg	0.001	0.001	0.9726	0.7801
YOLOv12-turbo-m-seg	0.002	0.01	0.9722	0.7782

Results in bold indicate the best performance.

**Table 9:** Phase 4: Optimization of Warmup and Scheduler. The proposed model is marked with ^†^.

Model	*lr*_0_	Warmup	Cosine LR	mAP50	mAP50-95
YOLOv12-turbo-m-seg ^†^	0.001	3	true	**0.9800**	0.7859
YOLOv11l-seg	0.002	1	true	0.9772	0.7844
YOLOv11-m-seg	0.0002	3	true	0.9763	0.78619
YOLOv8-m-seg	0.0002	3	true	0.9758	**0.7871**
YOLOv12-turbo-m-seg	0.002	1	true	0.9745	0.7821
YOLOv12-turbo-m-seg	0.001	3	false	0.9728	0.7759
YOLOv12-turbo-m-seg	0.002	5	true	0.9715	0.7783

Results in bold indicate the best performance.

**Table 10:** Evaluation of various optimizers with variation in the batch size.

Optimizer	Momentum	Batch	Notes	mAP50	mAP50-95
SGD	0.9	2	SGD on half batch	**0.9800**	0.7859
SGD	0.9	4	SGD Baseline	0.9788	0.7841
SGD	0.95	4	Higher momentum	0.9756	**0.7877**
Adam	0.999	4	Adam comparison	0.9743	0.7825
NAdam	0.999	4	NAdam test	0.9736	0.7849
RMSProp	0.999	4	RMSProp Test	0.9078	0.6053

Results in bold indicate the best performance.

**Table 11:** Evaluation of performance impact when varying the input image size resolution.

Optimizer	Batch	Image Size	mAP50	mAP50-95
SGD	2	1280	**0.9800**	0.7859
auto	4	1600	0.9786	**0.7978**
auto	4	1024	0.9763	0.7734
auto	4	640	0.9757	0.7151
auto	2	640	0.9743	0.7164
SGD	4	640	0.9722	0.7094

Results in bold indicate the best performance.

**Table 12:** Evaluation of different batch sizes with different optimizers.

Optimizer	*lr*_0_	Batch	mAP50	mAP50-95
SGD	0.001	2	**0.9800**	0.7859
SGD	0.001	4	0.9788	0.7841
SGD	0.001	8	0.9759	0.7850
auto	0.001	4	0.9766	**0.7896**
auto	0.001	8	0.9754	0.78618
AdamW	0.002	10	0.9753	0.7826
AdamW	0.002	4	0.9745	0.7821

Results in bold indicate the best performance.

**Table 13:** Evaluation of different weight decay values and regularization parameters.

Weight Decay	Close Mosaic	Patience	mAP50	mAP50-95
0.0005	10	30	**0.9800**	0.7859
0.001	10	30	0.9785	0.7889
0.0001	10	30	0.9773	**0.7903**
0.0005	5	50	0.9728	0.7831

Results in bold indicate the best performance.

**Table 14:** Evaluation of dropout regularization.

Dropout	Notes	mAP50	mAP50-95
0.0	Zero regularization	**0.9800**	0.7859
0.1	Light regularization	0.9766	**0.7896**
0.2	Medium regularization	0.9723	0.7850

Results in bold indicate the best performance.

**Table 15:** Multi-seed reproducibility analysis for the proposed segmentation model (YOLOv12-turbo-m-seg with PIoUv2 loss). All runs use the same hyperparameters and training/test split with different random seeds.

Metric	Seeds					Summary	
	123	456	789	1024	426	Mean ± std	CV (%)
mAP@50	0.9768	0.9756	0.9754	0.9761	0.9730	0.9754 ± 0.0014	0.15
mAP@50-95	0.7882	0.7893	0.7891	0.7894	0.7873	0.7887 ± 0.0009	0.11
Precision	0.9809	0.9857	0.9812	0.9857	0.9868	0.9840 ± 0.0028	0.28
Recall	0.9734	0.9702	0.9733	0.9708	0.9678	0.9711 ± 0.0023	0.24

CV = coefficient of variation.

## Data Availability

The datasets used in this study are publicly available and can be accessed through the references provided in the manuscript. The code used for the experiments can be made available upon reasonable request to the corresponding author.

## Abbreviations

The following abbreviations are used in this manuscript:

CBCT	Cone-Beam Computed Tomography
CEJ	Cemento-Enamel Junction
CLAHE	Contrast-Limited Adaptive Histogram Equalization
CNN	Convolutional Neural Network
DL	Deep Learning
FDI	Fédération Dentaire Internationale (FDI notation)
IoU	Intersection over Union
ML	Machine Learning
MBL	Marginal Bone Loss
NAdam	Nesterov-accelerated Adam
OPT	Orthopantomogram (panoramic radiograph)
PAI	Periapical Index
PBL	Periodontal Bone Loss
PIoUv2	Powerful IoU v2 (Loss)
RBL	Radiographic Bone Loss
RMSProp	RMSProp Optimizer
ROI	Region of Interest
SGD	Stochastic Gradient Descent
TTA	Test-Time Augmentation
YOLO	You Only Look Once (object detector family)
mAP	mean Average Precision
mDice	mean Dice coefficient
mIoU	mean Intersection over Union
